# Interaction of Neurovascular Signals in the Degraded Condylar Cartilage

**DOI:** 10.3389/fbioe.2022.901749

**Published:** 2022-04-29

**Authors:** Wenpin Qin, Zibin Zhang, Jianfei Yan, Xiaoxiao Han, Li-Na Niu, Kai Jiao

**Affiliations:** ^1^ State Key Laboratory of Military Stomatology and National Clinical Research Center for Oral Diseases and Shaanxi Key Laboratory of Stomatology, School of Stomatology, The Fourth Military Medical University, Xi’an, China; ^2^ The College of Life Science, Northwest University, Xi’an, China

**Keywords:** angiogenesis, temporomandibular disorders, bioinformatics, growth factor, chondrocyte

## Abstract

**Introduction:** Degradation of the condylar cartilage during temporomandibular joint osteoarthritis (TMJ-OA) results in the infiltration of nerves, blood vessels and inflammatory cells from the subchondral bone into the cartilage. The interaction among innervation, angiogenesis and inflammation in the condylar cartilage of TMJ-OA remains largely unknown.

**Method:** In the present study, microarray-based transcriptome analysis was used to detect, and quantitative real-time polymerase chain reaction was used to validate transcriptome changes in the condylar cartilage from a well-established rat TMJ-OA model. Gene ontology (GO), Kyoto encyclopedia of genes and genomes (KEGG) pathway and protein-protein interaction (PPI) analyses were conducted.

**Result:** There were 1817 differentially expressed genes (DEGs, fold change ≥2, *p* < 0.05) between TMJ-OA and control cartilages, with 553 up-regulated and 1,264 down-regulated genes. Among those genes, representative DEGs with known/suspected roles in innervation, angiogenesis and inflammation were further validated by enriched GO terms and KEGG pathways. The DEGs related to innervation were predominately enriched in the GO terms of neurogenesis, generation of neurons, and KEGG pathways of cholinergic synapse and neurotrophin signaling. Genes related to angiogenesis were enriched in GO terms of vasculature and blood vessel development, and KEGG pathways of hypoxia-inducible factor 1 (HIF-1) pathway and calcium signaling pathway. For inflammation, the DEGs were enriched in the GO terms of immune system process and immune response, and KEGG pathways of Toll-like receptor and transforming growth factor β (TGFβ) signaling. Analysis with PPI indicated that the aforementioned DEGs were highly-interacted. Several hub genes such as v-akt murine thymoma viral oncogene homolog 1 (*Akt1*), glycogen synthase kinase 3β (*Gsk3b*), fibroblast growth factor 2 (*Fgf2*) and nerve growth factor receptor (*Ngfr)* were validated.

**Conclusion:** The present study demonstrated, for the first time, that intimate interactions exist among innervation, angiogenesis and inflammation in the condylar cartilage of TMJ-OA.

## Introduction

Temporomandibular joint osteoarthritis (TMJ-OA) is characterized by progressive cartilage degradation, thickening of the calcified cartilage and subchondral bone changes. This condition is one of the most complex temporomandibular disorders (Chen et al., 2020). The osteochondral junction in the TMJ comprises a calcified cartilage layer and a subchondral plate. In normal joints, the terminal blood vessels in the subchondral plate are in direct contact with, or infiltrate the calcified cartilage. These vessels are rarely detected in the non-calcified cartilage beyond the tidemark (i.e., boundary between the calcified and non-calcified cartilage) (Wang et al., 2012). In OA, blood vessels and nerve endings from the subchondral bone can breach the tidemark and cause channels to extend from the subchondral bone into the non-calcified cartilage (Zhu et al., 2019). Pathological neurovascularization within the non-calcified cartilage damages the normal barrier between articular cartilage and subchondral bone. Infiltration of inflammatory cells from the blood vessels disrupts chondrocyte function and degrades the cartilage matrix (Findlay and Kuliwaba, 2016). Pathological neurovascularization is also an essential component of endochondral ossification (Wang et al., 2012). Alteration of the osteochondral junction further aggravates OA progression via direct involvement of innervation, angiogenesis, and inflammation (Jiao et al., 2015; Chen et al., 2020).

Angiogenesis and innervation are closely integrated processes in pathological conditions such as cancer and arthritis (Jin et al., 2020; Hu et al., 2021). In OA, perivascular nerve growth causes the sensory nerves within the cartilage to be exposed to chemical and mechanical stimulation, thereby contributing to osteoarthritic pain. Neuropeptides such as calcitonin gene-related peptide and substance p, in turn, act on specific vascular receptors to regulate blood flow and permeability, as well as stimulating endothelial cell proliferation, migration and tube formation to promote growth of the blood vessels. Sympathetic neuropeptides such as neuropeptide Y are also acute vasoconstrictors that increase blood flow and promote angiogenesis (Mapp and Walsh, 2012). Apart from the contribution of blood vessel growth to inflammation in arthritis, increased infiltration of macrophages also provides a key stimulus for angiogenesis through the production of proangiogenic factors such as vascular endothelial growth factor (VEGF). Nerve growth regulators are also produced by vascular cells and inflammatory cells in the perivascular environment (Marrella et al., 2018; Wan et al., 2021). Although the growth of blood vessels, nerves and the infiltration of inflammatory cells are closely interacting processes that share common regulatory pathways, information on the interactions of these three processes in the TMJ-OA cartilage is scanty (Marrella et al., 2018).

Previous work by the authors demonstrated that OA-like lesions were created by unilateral anterior crossbite (UAC)-induced dysregulated mechanical loading of rodent TMJs (Sun J.-l. et al., 2020; Sun et al., 2020 J. L.). In the present study, pathologic changes in the blood vessels and nerves from the osteochondral junction to condylar cartilage of UAC mice were characterized. Microarray-based transcriptome analysis was used to select target genes involved in innervation, angiogenesis and inflammation in the cartilages of UAC mice and the corresponding control. Enrichment analysis and protein-protein interaction analysis were used to identify differentially expressed genes (DEGs) that were associated with these processes. The hypothesis to be tested was that there is intricate crosstalk among innervation, angiogenesis and inflammation during OA progression.

## Materials and Methods

Male Sprague-Dawley rats (140–160 g; 6-week-old) were obtained from the animal center of the Air Force Medical University (AFMU), Xi’an, China. Animal experimental protocols were reviewed and approved by the Institutional Animal Care and Use Committee of AFMU, following the National Institute of Health Guidelines for the Care and Use of Laboratory Animals. The procedures complied with ARRIVE 2.0 guidelines. Unilateral anterior crossbite appliances (metal tubes) were fitted to the jaws of the rats to induce osteoarthritic-like changes in their TMJ cartilage (Jiao et al., 2016). In the control group, rats were subjected to the aforementioned procedures except for the bonding of the UAC appliances.

### Histochemical and Immunohistochemical Staining

All rats were euthanized with pentobarbital overdose. A 3 × 4 mm tissue block that included the joint capsule and surrounding soft tissue was harvested gently from each rat. For hematoxylin-eosin and Safranin O/Fast Green staining, the tissue blocks were fixed in 4% paraformaldehyde, decalcified in 10% ethylenediamine tetra-acetic acid and embedded in paraffin. The TMJ was sectioned in the sagittal plane. The sections were mounted on poly-L-lysine-coated glass slides and deparaffinized. Half of the sections were stained with hematoxylin and eosin. For Safranin O/Fast Green staining, the sections were stained with 0.02% Fast Green and subsequently with 0.1% safranin O. The central sagittal sections of each TMJ section were used for examination.

For immunofluorescence staining, the specimens were sequentially fixed in 4% paraformaldehyde and 30% sucrose, embedded in optimal cutting temperature compound (Leica, Wetzlar, Germany) and stored at −80 C. After blocked with 1.5% goat serum (MilliporeSigma, Burlington, MA), the sections were incubated with CGRP (ab36001, Abcam, Cambridge, United Kingdom) or CD31 (sc-376764, Santa Cruz Biotechnology, Dallas, TX) primary antibodies and subsequently with secondary antibody (US Everbright Inc., Suzhou, China). The sections were rinsed with phosphate-buffered saline then mounted with Prolong Diamond Antifade Mountant containing 4’,6-diamidino-2-phenylindole (DAPI; Invitrogen, San Diego, CA). Images were acquired using laser scanning confocal microscopy (FV1000, Olympus, Tokyo, Japan).

### Microarray

Condylar cartilages were ground in liquid nitrogen. Total RNA was extracted using Trizol reagent and purified using a RNAeasy mini kit (Qiagen, Hilden, Germany) according to the manufacturer’s instructions. All specimens were treated on-column with DNase. The isolated total RNA was translated into cDNA and labeled with Cy3 using the Quick Amp Labeling kit (Agilent Technologies, Santa Clara, CA). After matched with the same concentration of RNA, the specimens were prepared for Whole Rat Genome Microarray (4 × 44K; Agilent Technologies). The hybridization signal intensities were detected using a microarray scanner (G2505C, Agilent). Signals were read, normalized and analyzed using the Feature Extraction 9.5.3 and GeneSpring GX10 software (Agilent). The DEGs were identified as fold change ≥2 (*p* < 0.05). Analyses with Gene Ontology (GO) terms and Kyoto Encyclopedia of Genes and Genomes (KEGG) pathway (Kanehisa et al., 2019) were undertaken by the Database for Annotation, Visualization and Integrated Discovery (DAVID; Laboratory of Immunopathogenesis and Bioinformatics, SAIC, Frederick, Inc., Frederick, MD). Analysis of protein-protein interaction (PPI) was undertaken by Search Tool for the Retrieval of Interacting Genes/Proteins (STRING; https://string-db.org/) (Szklarczyk et al., 2019) and CytoScape (version 3.9.0, Institute of Systems Biology, Seattle, WA; https://cytoscape.org/) (Shannon et al., 2003).

### Quantitative Real-Time Polymerase Chain Reaction

Quantitative real-time polymerase chain reaction (qRT-PCR) was performed as previously reported (Ma et al., 2022). The following genes were used for qRT-PCR: v-akt murine thymoma viral oncogene homolog 1 (*Akt1*), glycogen synthase kinase 3β (*Gsk3b*), fibroblast growth factor 2 (*Fgf2*) and nerve growth factor receptor (*Ngfr)*. Glyceraldehyde-3-phosphate dehydrogenase (*Gapdh*) was used as the housekeeping gene for data normalization using the 2^–∆∆Ct^ method. GraphPad Prism 8.0 (GraphPad Software, San Diego, CA) was used to examine if significant difference in fold expressions of the tested genes existed between the UAC group and the control group. The Student’s t test was used for statistical analysis. Statistical significance was preset at α = 0.05. Because the four genes tested were derived from the same rat genome, the t-tests performed belonged to the same “family” of statistical procedures. The Bonferroni correction was used to control the familywise error rate to reduce the chance of obtaining false-positive conclusions. Accordingly, *p* values less than 0.05/4 = 0.013 were considered significantly different for comparison of fold changes of the four genes between the UAC and control groups.

## Results

### Validation of Cartilage Changes in Osteoarthritis

**FIGURE 1:**
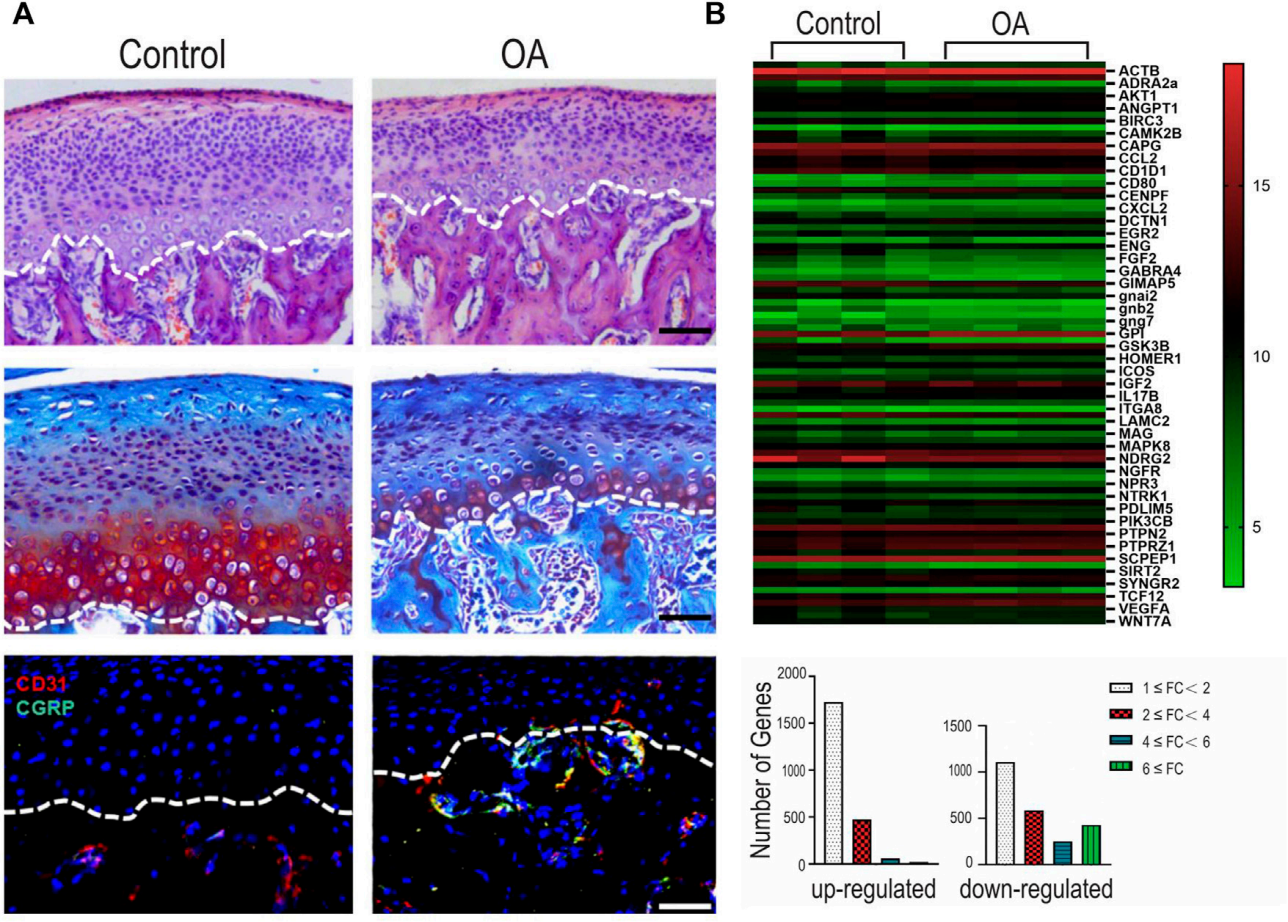
**(A)** Representative images of hematoxylin-eosin (HE) staining, Safranin O/Fast Green staining and immunofluorescence staining of the mandibular condylar cartilage in the fibrocartilages harvested from the unilateral anterior crossbite (UAC) group versus the control group. Bar = 50 µM. **(B)** Integrated analyses of differentially expressed genes (DEGs). Top: heatmap. The horizontal axis represents cluster analysis of groups. The vertical axis represents cluster analysis of genes. Red represents up-regulated DEGs, green represents down-regulated DEGs, black represents gene expression with no difference. The degree of significant difference in gene expression is indicated by the brightness of the color. Bottom: DEGs were classified according to the differential expression level with a minimum of 1-fold, 2-fold, 4-fold, and 6-fold differences. FC: fold change.

Histochemical and immunohistochemical staining were used to characterize the rat TMJ-OA model (Figure 1A). The condylar cartilage appeared normal in the control group, with four intact layers. The cartilage had an overall smooth surface and contained homogenously-distributed chondrocytes. The UAC group manifested OA-like changes such as the loss of cartilage surface integrity, abnormal thickening of the four cartilage layers and cellular derangement. There was no significant difference in the changes within the right and left condylar cartilage in the UAC group. Pathological neurovascularization, in the form of new erythrocyte-containing blood vessels and nerves, infiltrated the osteochondral junction into the non-calcified condylar cartilage in the TMJ-OA rat model.

### Differential Gene Expression in the Condylar Cartilage

Of the 4,650 genes detectable using the Whole Rat Genome Microarray, 1817 significant DEGs with fold change ≥2 (*p* < 0.05) were detected in the condylar cartilage of the UAC group. Among the DEGs, 553 genes were up-regulated and 1,264 genes were down-regulated. Specific DEGs related to innervation, angiogenesis and inflammation are depicted in Supplementary Appendix Table S1–S4, respectively. The heatmap and bar chart in Figure 1B showed the fold changes in selected genes expressed by the condylar cartilage in the UAC group versus the control group.

### Enrichment Analysis of Differential Gene Expression

**FIGURE 2:**
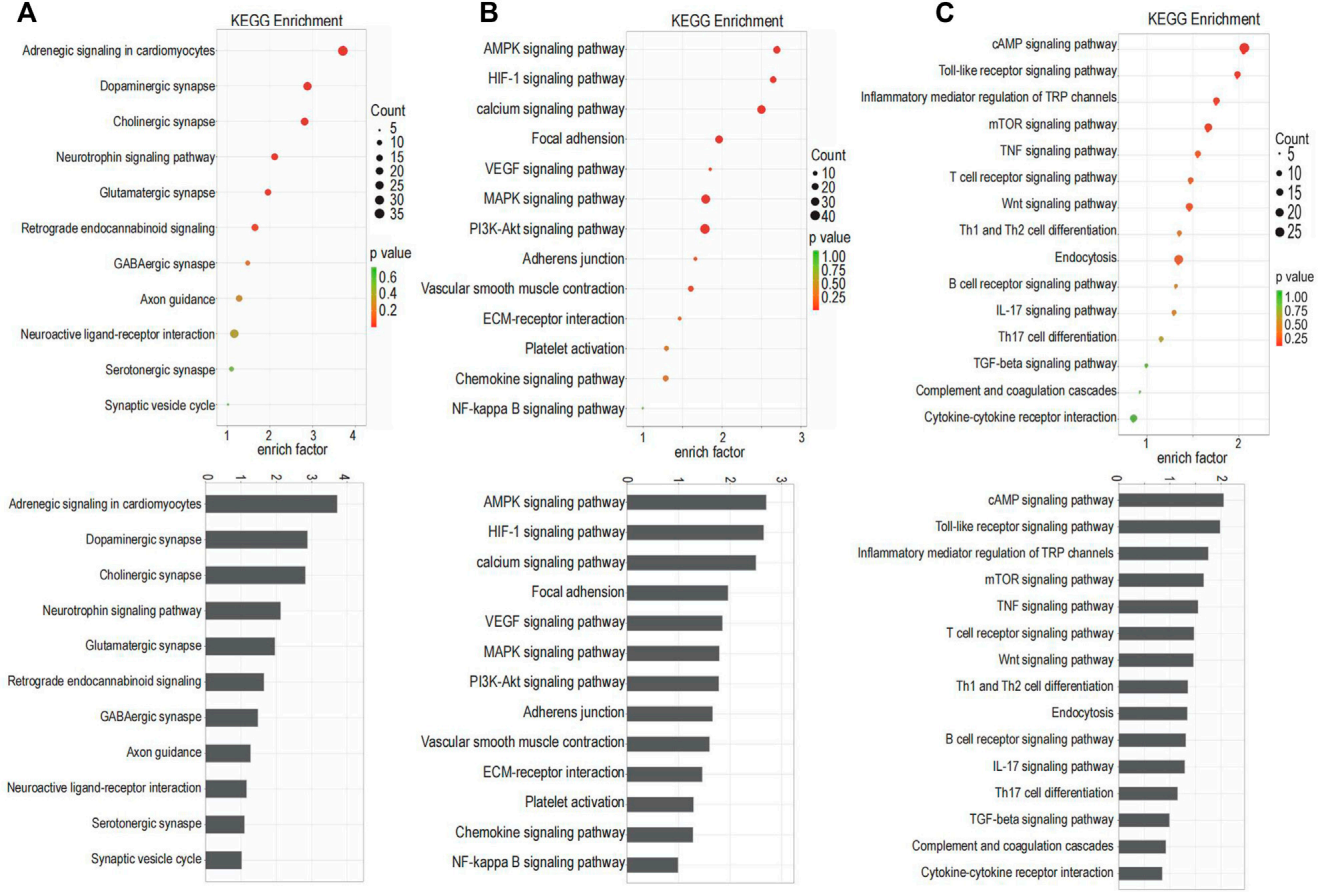
The Kyoto Encyclopedia of Genes and Genomes (KEGG) pathways enriched for the targeted genes. **(A)** The KEGG pathways related to innervation. **(B)** The KEGG pathways related to angiogenesis. **(C)** The KEGG pathways related to inflammation.

**FIGURE 3:**
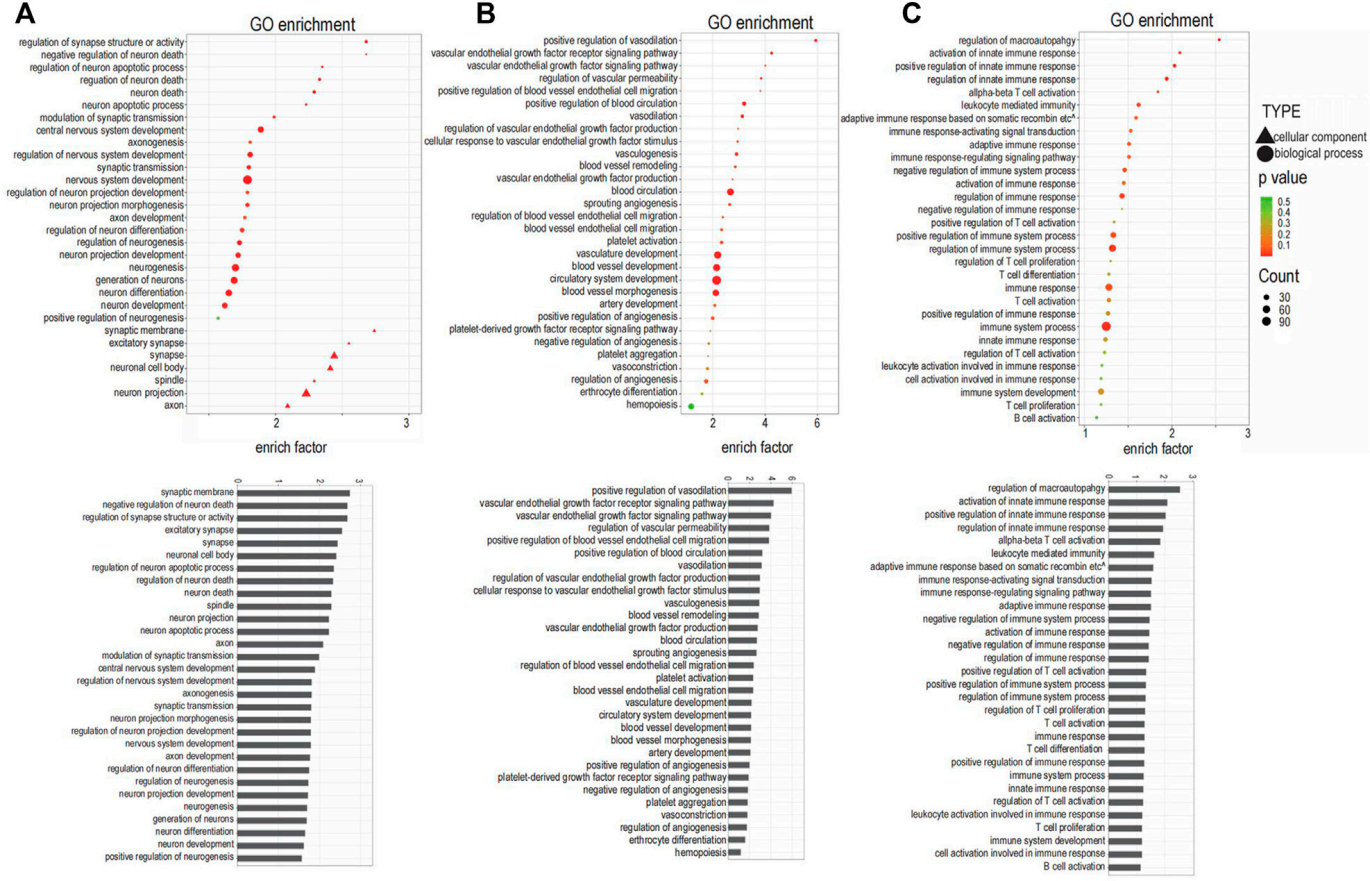
Gene Ontology (GO) analysis enriched for the targeted genes. **(A)** GO terms related to innervation. **(B)** GO terms related to angiogenesis. **(C)** GO terms related to inflammation.

GO term enrichment and KEGG pathway analysis were performed on innervation-related, angiogenesis-related and inflammation-related genes to obtain more detailed information on correlated pathways and their potential functions. The KEGG pathways (Figure 2) and GO terms (Figure 3) related to innervation, angiogenesis and inflammation are presented in the order of enriched factor. Genes related to innervation were enriched in several classical KEGG pathways such as dopaminergic synapse, cholinergic synapse and neurotrophin signaling pathway (Figure 2A). Genes related to angiogenesis were enriched in the AMP-activated protein kinase (AMPK), hypoxia-inducible factor (HIF)-1 and calcium signaling pathways (Figure 2B). Genes related to inflammation were enriched in the cationic antimicrobial peptide (cAMP) signaling pathway, Toll-like receptor signaling pathway and inflammatory mediator regulation of transient receptor potential (TRP) channels (Figure 2C).

The top three enriched GO terms related to innervation were regulation of synapse structure or activity, negative regulation of neuron death, and regulation of neuron apoptotic process; additional enriched terms are depicted in Figure 3A. The top three enriched GO terms related to angiogenesis were positive regulation of vasodilation, VEGF receptor signaling pathway, and VEGF signaling pathway; additional enriched terms are depicted in Figure 3B. The top three enriched GO terms related to inflammation were regulation of macroautophagy, activation of innate immune response, and positive regulation of innate immune response; additional enriched terms are depicted in Figure 3C.

Among the 30 GO terms related to innervation, 12 terms were related to synapse and axons, suggesting that neurotransmitters are involved in the extension of neurons into the condylar cartilage. Angiogenesis was not only closely related to the angiogenic factors but was related also to classical signaling pathways including AMPK, MAPK, PI3K-Akt, and NF-kappa B signaling. The enriched GO terms related to inflammation identified a possible role for T cells, based on their frequency of occurrence.

### Protein-Protein Interaction (PPI) Network Analysis of DEGs

**FIGURE 4:**
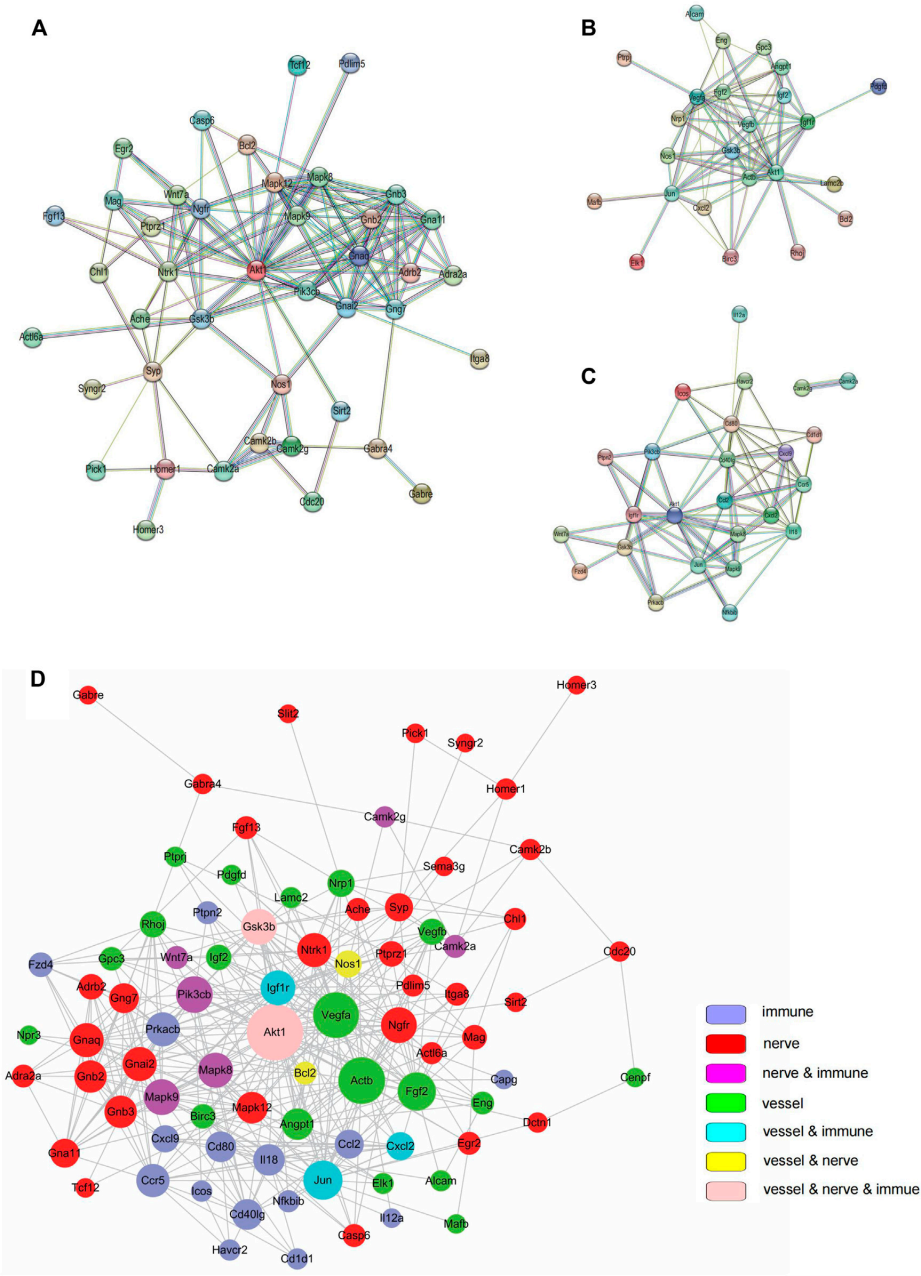
Protein-protein interaction (PPI). **(A)** PPI among genes related to innervation. **(B)** PPI angiogenesis related to angiogenesis. **(C)** PPI among genes related to inflammation. **(D)** PPI among all targeted genes. In **(A–C)**, PPIs were determined with STRING software. In **(D)**, PPI was determined using the Cytoscape software.

The STRING database and Cytoscape were to analyze PPI to detect hub genes (i.e., genes with high correlation in candidate modules) among the selected genes. The innervation-related genes (Figure 4A), angiogenesis-related genes (Figure 4B) and inflammation-related genes (Figure 4C) were first analyzed separately. All selected genes were then analyzed together to further validate their interactions (Figure 4D). Different colors on the dot represented different functions. Different dot sizes represented closeness (i.e., the larger the point, the closer the relationship). The interrelated genes identified included *Akt1*, *Igf1r*, *Vegfa*, *Pik3cb*, *Ngfr*, *Mapk8*, *Mapk12*, *Actb*, *Fgf2*, *Gsk3b*, *Ccl2,* and *Jun*. Remarkably, *Akt1* and *Gsk3b* participated in all the three processes - innervation, angiogenesis and inflammation. *Akt1*, *Igf1r* and *Vegfa* shared several pathways, including PI3K-Akt signaling pathway, MAPK signaling pathway and HIF-1 signaling pathway. These results revealed the strong interactions among innervation, angiogenesis and inflammation in the TMJ-OA cartilage.

### qRT-PCR

**FIGURE 5:**
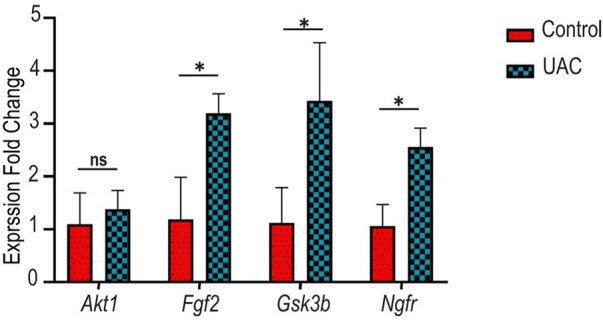
Gene expression patterns of v-akt murine thymoma viral oncogene homolog 1 (*Akt1*), fibroblast growth factor 2 (*Fgf2*), glycogen synthase kinase 3 beta (*Gsk3b*) and nerve growth factor receptor (*Ngfr*). Evaluated was performed with quantitative real-time polymerase chain reaction (qRT-PCR). ns indicates *p* > 0.013 (after Bonferroni correction of familywise error rate, * indicates *p* < 0.013. Analyses were performed using paired t-tests.

To confirm the findings of the microarray analysis, qRT-PCR was conducted on four genes involved in innervation, angiogenesis and inflammation (*Akt1*, *Fgf2*, *Gsk3b*, *Ngfr*). The expression profiles of these genes had a good match for the results obtained by microarray analysis (Figure 5). Minor differences were attributed to the heterogeneity of OA between individual animals. The qRT-PCR results validated the accuracy of the microarray data and supported that changes in innervation-related, angiogenesis-related and inflammation-related genes were detected in the condylar cartilage of OA rats.

## Discussion

Neurovascularization within the non-calcified cartilage is a critical pathological change in OA (Mapp and Walsh, 2012; Li B. et al., 2021). In the present study, pathological neurovascularization was validated in TMJ-OA cartilage. Microarray analysis of genes from the condylar cartilage of TMJ identified 1817 significant DEGs between the control and UAC groups. Based on their functions, target genes involved in innervation, angiogenesis and inflammation were selected for further analyses. After enrichment analysis and PPI analysis, the robust interactions among innervation, angiogenesis and inflammation were confirmed in the condylar cartilage of TMJ-OA. Several other microarray analyses about osteoarthritis cartilage also supported our findings. One of which was carried out on IL-1β-stimulated chondrocytes to identify the potential biological processes in the progression of osteoarthritis (Liang et al., 2022). Similarly, they found DEGs were mainly enriched in these terms, including “inflammatory response”, “blood vessel morphogenesis”, “epithelial cell proliferation” and so on. However, different from our results, the terms about neurogenesis were not included in that research, which may because the subject of our research was cartilage from animal while that of their research was cultured chondrocyte. Another analysis about OA cartilage also found the changes in PI3K/Akt signaling pathway played an important role, which supporting our results (Li S. et al., 2021).

The contribution of angiogenesis to OA progression has been investigated extensively. Osteochondral angiogenesis was observed in mandibular condyles with OA-like changes, with concomitant local up-regulation of VEGF, connective tissue growth factor and matrix metalloprotease-9 (Wang et al., 2012). Growth factors such as Vegfa, Vegfb, Pdgf, and Angpt1 are promoters of angiogenesis (MacDonald et al., 2018); these factors were up-regulated in OA cartilage and down-regulated in the control. Both VEGFa and VEGFb are members of the VEGF family. These angiogenesis regulators act on endothelial cells and pericytes around blood vessels. Platelet-derived growth factor (PDGF), a pro-angiogenic factor derived from preosteoclasts in the subchondral bone, directly induces proliferation of endothelial cells to form type H vessels and stimulates VEGF secretion (Su et al., 2020). Angiopoietin 1 (ANGPT1) is a vascular growth factor that controls endothelial sprouting by regulating vascular stability. It interacts with tyrosine-protein kinase receptor 2 (TIE2) to activate several signaling pathways, including the PI3K/AKT pathway, to ensure endothelial survival and junctional stability (Saharinen et al., 2017). With the use of KEGG analysis, the MAPK, HIF-1 and nuclear factor-kappa B (NF-κB) signaling pathways were significantly enriched in the DEGs. Regulated by the MAPK and NF-κB pathways, HIF-1α upregulates VEGFa expression and promotes type H vessel formation (Fernandez et al., 2017). Hence, the present data confirm that angiogenesis is involved in TMJ-OA progression. Because angiogenesis contributes to synovitis, osteochondral damage, and osteophyte formation in patients with TMJ-OA, inhibition of angiogenesis is potentially useful for treatment of TMJ-OA.

Infiltration of sensory nerves and sympathetic nerves through the osteochondral junction into cartilage occurs after inflammation and angiogenesis in TMJ-OA (Zylinska et al., 2021). Subchondral bone deterioration through increased bone remodeling and nerve sprouting has been identified in the osteochondral junction of osteoarthritic joints and is closely associated with osteoarthritic pain in OA rats (Sun J.-l. et al., 2020). Although the role of sensory nerves and sympathetic nerves in OA has been well demonstrated, pathological innervation in TMJ-OA cartilage requires further studies. The present study demonstrated up-regulation of *Ngfr* in the condylar cartilage of TMJ-OA. This factor induces pain through amplified proliferation of sensory neurons that alter the pain threshold (Jiang et al., 2015). Others reported that gene deletion of NGF produced pain-palliative effects (Zhao et al., 2019). Apart from innervation, NGF/NGFR also stimulates the expression of VEGF and promotes vascular growth. This accounts for the link between angiogenesis and innervation in TMJ-OA. Examination of articular cartilages harvested from healthy and OA patients identified that NGF increased angiogenesis by promoting the expression of FGF2 via PI3K/Akt and ERK/MAPK signaling pathways (Yu et al., 2019). Identification of the up-regulation of *Adrb2* and *Adra2a* genes in the UAC group is indicative of the penetration of sympathetic nerve fibers into the cartilage during TMJ deterioration. Further studies are needed to confirm this finding and clarify the role of pathological innervation in TMJ-OA cartilage.

Because the interactions among innervation, angiogenesis and inflammation contribute to structural damage and pain in TMJ-OA, interception of these interactions offer potential conduits for relieve of symptoms and prevent joint pathology in TMJ-OA. For example, inhibiting inflammation and reducing cartilage innervation produce direct symptomatic benefits for OA patients. Inhibition of angiogenesis reduces pain by moderating each of these pathological processes. Apart from targeting the nervous system, potential nano-therapies that target blood vessels or inflammation also help to relieve pain symptoms (Li X. et al., 2021). Although treatment strategies that individually target angiogenesis (Li et al., 2019; Yajun et al., 2021), pain or inflammation (Lee et al., 2014; Bhalekar et al., 2016) are casually effective in alleviating OA symptoms, therapies that explicitly target the crosstalk among innervation, angiogenesis and inflammation may potentially become the therapeutic target of the future.

The gene expression profiles of TMJ-OA identified in the present study may not completely match those reported by other studies. This may be attributed to the limitations inherent in the present study. These results we obtained were merely based on limited sample size (four in each group), leading to difficulty in excluding false positive. With the development of scientific technology, bulk RNA-sequencing, single-cell RNA-sequencing and spatial transcriptomics have become feasible over the last few years. These timely techniques have become extremely popular assays for transcriptome measurements. In the future, high-throughput sequencing technology should be applied to examine the mechanism of TMJ-OA. It is imperative to mention that differences exist between animal models and human versions of TMJ-OA. Results obtained from animal models should be tested prior to their translation for treatment of human diseases.

In conclusion, the highly interactive crosstalk among innervation, angiogenesis and inflammation in the condylar cartilage is a contributor to disease progression in TMJ-OA. This crosstalk offers possible directions for future research on therapeutics to address TMJ-OA.

## Data Availability

The datasets presented in this study can be found in online repositories. The names of the repository/repositories and accession number(s) can be found in the article/Supplementary Material.
