# Supplementary material for: Interaction of Neurovascular Signals in the Degraded Condylar Cartilage
Source: Front Bioeng Biotechnol. 2022 Apr 29;10:901749. doi: 10.3389/fbioe.2022.901749 (PMC9099211; doi:10.3389/fbioe.2022.901749)
Supplement: Supplementary file 1 [file DataSheet1.docx]

Appendix Table 1. Representative differentially expressed genes (DEGs) with known or suspected roles in innervation.

| **Gene symbol** | **Description** | **p value** | **fold change** | **up/down-regulation** |
| --- | --- | --- | --- | --- |
| Fgf13 | Fibroblast growth factor 13 | 0.0356 | 14.04 | down |
| Camk2b | Calcium/calmodulin dependent protein kinase II β | 0.0094 | 12.51 | down |
| Ndrg2 | NDRG family member 2 | 0.0053 | 9.02 | down |
| Gpr83 | G protein-coupled receptor 83 | 0.0298 | 7.98 | down |
| Pdlim5 | PDZ and LIM domain 5 | 0.0264 | 6.86 | down |
| Adar2a | Adrenoceptor α 2A | 0.0236 | 5.12 | up |
| Gnb2 | G protein subunit β 2 | 0.0220 | 5.11 | up |
| Ptprz1 | Protein tyrosine phosphatase receptor type Z1 | 0.0005 | 4.88 | up |
| Homer1 | Homer scaffold protein 1 | 0.0332 | 4.72 | down |
| Gna11 | G protein subunit α 11 | 0.0149 | 4.63 | up |
| Adrb2 | Adrenoceptor β 2 | 0.0368 | 4.51 | up |
| Itga8 | Integrin subunit α 8 | 0.0089 | 4.51 | down |
| Camk2g | Calcium/calmodulin dependent protein kinase II γ | 0.0123 | 4.47 | down |
| Gnb3 | G protein subunit β 3 | 0.0027 | 4.45 | up |
| Ache | Acetylcholinesterase | 0.0082 | 4.40 | down |
| Chl1 | Cell adhesion molecule L1 like | 0.0093 | 4.32 | up |
| Gnaq | G protein subunit α q | 0.0027 | 4.30 | up |
| Tcf12 | Transcription factor 12 | 0.0097 | 4.13 | up |
| Dctn1 | Dynactin subunit 1 | 0.0170 | 3.98 | down |
| Sema3g | Semaphoring 3G | 0.0211 | 3.97 | down |
| Sirt2 | Sirtuin 2 | 0.0319 | 3.92 | down |
| Nos1 | Nitric oxide synthase 1 | 0.0300 | 3.88 | down |
| Gabra4 | γ-aminobutyric acid type A receptor subunit α4 | 0.0169 | 3.86 | down |
| Mapk12 | γ-activated protein kinase 12 | 0.0447 | 3.81 | down |
| Gabre | γ-aminobutyric acid type A receptor subunit epsilon | 0.0351 | 3.71 | down |
| Slit2 | Slit guidance ligand 2 | 0.0089 | 3.65 | up |
| Casp6 | Caspase 6 | 0.0165 | 3.59 | up |
| Mapk9 | Mitogen-activated protein kinase 9 | 0.0162 | 3.59 | up |
| Ngfr | Nerve growth factor receptor | 0.0249 | 3.58 | up |
| Akt1 | AKT serine/threonine kinase 1 | 0.0177 | 3.50 | up |
| Syngr2 | Synaptogyrin 2 | 0.0014 | 3.45 | down |
| Ntrk1 | Neurotrophic receptor tyrosine kinase 1 | 0.0345 | 3.43 | down |
| Cdc20 | Cell division cycle 20 | 0.0295 | 3.42 | up |
| Bcl2 | B cell leukemia/lymphoma 2 | 0.0245 | 3.39 | down |
| Pick1 | Protein interacting with PRKCA 1 | 0.0252 | 3.37 | down |
| Syp | Synaptophysin | 0.0063 | 3.36 | up |
| Gsk3b | Glycogen synthase kinase 3 β | 0.0024 | 3.35 | up |
| Homer3 | Homer scaffold protein 3 | 0.0227 | 3.35 | up |
| Mapk8 | Mitogen-activated protein kinase 8 | 0.0187 | 3.34 | up |
| Actl6a | Actin like 6A | 0.0485 | 3.34 | up |
| Wnt7a | Wnt family member 7A | 0.0367 | 3.33 | down |
| Pik3cb | Phosphatidylinositol-4,5-bisphosphate 3-kinase catalytic subunit β | 0.0091 | 3.30 | up |
| Gnai2 | G protein subunit α i2 | 0.0216 | 3.23 | up |
| Camk2a | Calcium/calmodulin dependent protein kinase II α | 0.0021 | 3.14 | up |
| Egr2 | Early growth response 2 | 0.0346 | 3.04 | up |
| Gng7 | G protein subunit γ 7 | 0.0116 | 3.01 | up |

Appendix Table 2. Representative DEGs with known or suspected roles in angiogenesis.

| **Gene symbol** | **Description** | **p value** | **fold change** | **up/down**  **regulation** |
| --- | --- | --- | --- | --- |
| Gpc3 | Glypican 3 | 0.0101 | 8.26 | down |
| Igf2 | Insulin like growth factor 2 | 0.0416 | 4.76 | down |
| Cenpf | Centromere protein F | 0.0246 | 4.65 | up |
| Rhoj | Ras homolog family member J | 0.0340 | 4.43 | down |
| Lamc2 | Laminin subunit γ 2 | 0.0406 | 4.23 | down |
| Gpi | Glucose-6-phosphate isomerase | 0.0031 | 4.20 | down |
| Actb | Actin, β | 0.0103 | 4.07 | down |
| Vegfa | Vascular endothelial growth factor A | 0.0137 | 4.03 | up |
| Elk1 | ETS transcription factor ELK1 | 0.0252 | 4.02 | down |
| Jun | Jun proto-oncogene, AP-1 transcription factor subunit | 0.0148 | 3.98 | up |
| Fgf2 | Fibroblast growth factor 2 | 0.0436 | 3.96 | up |
| Dmtf1 | Cyclin D binding myb-like transcription factor 1 | 0.0055 | 3.90 | up |
| Nos1 | Nitric oxide synthase 1 | 0.0300 | 3.88 | down |
| Eng | Endoglin | 0.0469 | 3.88 | down |
| Pdgfd | Platelet derived growth factor D | 0.0243 | 3.85 | up |
| Npr3 | Natriuretic peptide receptor 3 | 0.0251 | 3.79 | down |
| Alcam | Activated leukocyte cell adhesion molecule | 0.0306 | 3.79 | up |
| Ptprj | Protein tyrosine phosphatase, receptor type, J | 0.0170 | 3.76 | up |
| Birc3 | Baculoviral IAP repeat-containing 3 | 0.0063 | 3.67 | up |
| Cxcl2 | C-X-C motif chemokine ligand 2 | 0.0114 | 3.50 | up |
| Akt1 | AKT serine/threonine kinase 1 | 0.0177 | 3.50 | up |
| Nrp1 | Neuropilin 1 | 0.0485 | 3.47 | up |
| Bcl2 | B cell leukemia/lymphoma 2 | 0.0245 | 3.39 | down |
| Mafb | MAF bZIP transcription factor B | 0.0248 | 3.38 | down |
| Vegfb | Vascular endothelial growth factor B | 0.0390 | 3.36 | up |
| Gsk3b | Glycogen synthase kinase 3 β | 0.0024 | 3.35 | up |
| Igf1r | Insulin-like growth factor 1 receptor | 0.0073 | 3.32 | down |
| Scpep1 | Serine carboxypeptidase 1 | 0.0431 | 3.29 | up |
| Angpt1 | Angiopoietin 1 | 0.0051 | 3.27 | up |

Appendix Table 3. Representative DEGs with known or suspected roles in inflammation.

| **Gene symbol** | **Description** | **p value** | **fold**  **change** | **up/down**  **regulation** |
| --- | --- | --- | --- | --- |
| Cd40lg | CD40 ligand | 0.0238 | 4.79 | up |
| Camk2g | Calcium/calmodulin dependent protein kinase II γ | 0.0123 | 4.47 | down |
| Fzd4 | Frizzled class receptor 4 | 0.0170 | 4.33 | down |
| Icos | Inducible T-cell co-stimulator | 0.0449 | 4.30 | up |
| Ccl2 | C-C motif chemokine ligand 2 | 0.0190 | 4.04 | up |
| Jun | Jun proto-oncogene, AP-1 transcription factor subunit | 0.0148 | 3.98 | up |
| Havcr2 | Hepatitis A virus cellular receptor 2 | 0.0127 | 3.95 | up |
| Nfkbib | NFKB inhibitor β | 0.0092 | 3.69 | down |
| Ptpn2 | Protein tyrosine phosphatase, non-receptor type 2 | 0.0387 | 3.65 | up |
| Cd80 | Cd80 molecule | 0.0377 | 3.65 | up |
| Il17b | Interleukin 17B | 0.0361 | 3.61 | down |
| Cd1d1 | CD1d1 molecule | 0.0496 | 3.60 | up |
| Mapk9 | Mitogen-activated protein kinase 9 | 0.0162 | 3.59 | up |
| Ccr5 | C-C motif chemokine receptor 5 | 0.0163 | 3.57 | up |
| Gimap5 | GTPase, IMAP family member 5 | 0.0340 | 3.51 | down |
| Cxcl2 | C-X-C motif chemokine ligand 2 | 0.0114 | 3.50 | up |
| Akt1 | AKT serine/threonine kinase 1 | 0.0177 | 3.50 | up |
| Il12a | Interleukin 12a | 0.0252 | 3.47 | up |
| Tnfsf13 | TNF superfamily member 13 | 0.0035 | 3.43 | up |
| Cxcl9 | C-X-C motif chemokine ligand 9 | 0.0336 | 3.39 | down |
| Il18 | Interleukin 18 | 0.0012 | 3.39 | up |
| Prkacb | Protein kinase cAMP-activated catalytic subunit β | 0.0021 | 3.39 | up |
| Capg | Capping actin protein, gelsolin like | 0.0192 | 3.38 | down |
| Gsk3b | Glycogen synthase kinase 3 β | 0.0024 | 3.35 | up |
| Mapk8 | Mitogen-activated protein kinase 8 | 0.0187 | 3.34 | up |
| Wnt7a | Wnt family member 7a | 0.0367 | 3.33 | down |
| Igf1r | Insulin-like growth factor 1 receptor | 0.0073 | 3.32 | down |
| Pik3cb | Phosphatidylinositol-4,5-bisphosphate 3-kinase catalytic subunit β | 0.0091 | 3.30 | up |
| Camk2a | Calcium/calmodulin dependent protein kinase II α | 0.0021 | 3.14 | up |

Appendix Table 4. Primer sequences used for RT-PCR in the present study.

| **Gene name** | **Forward/ Reverse** | **Sequence （5’-3’）** |
| --- | --- | --- |
| v-akt murine thymoma viral oncogene homolog 1 | Forward | TGAAGCTACTGGGCAAGGG |
|  | Reverse | GGTCGTGGGTCTGGAATGAG |
| Fibroblast Growth Factor 2 | Forward | CGAACCGGTACCTGGCTATGA |
|  | Reverse | GTATTTCCGTGACCGGTAAGTGTTG |
| Glycogen Synthase Kinase 3 β | Forward | ATCCTTATCCCTCCTCACGC |
|  | Reverse | TTATTGGTCTGTCCACGGTCTC |
| Nerve Growth Factor Receptor | Forward | ACCTCATTCCTGTCTATTGCTCC |
|  | Reverse | GCGCCTTGTTTATTTTGTTTGC |
